# Effects of extended stance time on a powered knee prosthesis and gait symmetry on the lateral control of balance during walking in individuals with unilateral amputation

**DOI:** 10.1186/s12984-019-0625-6

**Published:** 2019-11-29

**Authors:** Andrea Brandt, He ( Helen) Huang

**Affiliations:** 1 Joint Department of Biomedical Engineering, North Carolina State University, Raleigh, NC 27606 USA; 2 The University of North Carolina at Chapel Hill, Chapel Hill, NC 27599 USA

**Keywords:** Gait, Amputation, Prosthetic knee component, Balance, Visual feedback

## Abstract

**Background:**

Individuals with lower limb amputation commonly exhibit large gait asymmetries that are associated with secondary health issues. It has been shown that they are capable of attaining improved temporal and propulsive symmetry when walking with a powered knee prosthesis and visual feedback, but they perceive this pattern of gait to be more difficult. Rather than improving the efficiency of gait, improved gait symmetry may be increasing individuals’ effort associated with maintaining lateral balance.

**Methods:**

In this study, we used a simple visual feedback paradigm to increase the prosthesis-side stance time of six individuals with unilateral TFA or KD as they walked on a powered knee prosthesis at their self-selected speed. As they walked more symmetrically, we evaluated changes in medial-lateral center-of-mass excursion, lateral margin of stability, stride width, and hip abductor activity.

**Results:**

As the subjects increased their prosthesis-side stance time, their center-of-mass excursion and hip abductor activity significantly increased, while their lateral margin of stability significantly decreased on the prosthesis-side only. Stride width remained relatively unchanged with testing condition.

**Conclusions:**

Extended stance time on a powered knee prosthesis (yielding more symmetric gait) challenged the lateral balance of individuals with lower limb amputation. Lateral stability may be a reason they prefer an asymmetric gait, even with more advanced technology. Hip muscular changes post-amputation may contribute to the decline in stability on the prosthesis side. Interventions and advancements in prosthesis control aimed at improving their control of lateral balance may ameliorate the difficulty in walking with improved gait symmetry.

## Introduction

For individuals with transfemoral amputation (TFA) or knee disarticulation (KD), gait asymmetry (i.e. favoring the intact limb over the amputated limb) can have serious implications for the health of the intact limb [1–3]. Often, gait asymmetry is attributed to the lack of functionality of traditional, energetically-passive prosthetic knee components (herein referred to as passive knee prostheses) compared to the biological limb. These passive prostheses are often classified as mechanical or microprocessor (e.g. active) prostheses, but they cannot generate power. Modern, powered prosthetic knee components (herein referred to powered knee prostheses) are able to generate a wide range of joint mechanics and mimic biological knee motion, enabling individuals with TFA or KD to walk with greater prosthetic knee function compared to traditional passive prostheses [4]. Yet, these individuals continue to walk with considerably reduced stance time [5] and ground reaction forces [6] on the prosthetic limb compared to the intact limb.

In our previous work, we demonstrated that five individuals with TFA or KD walking with a powered knee prosthesis and visual cueing could generate larger anterior propulsive forces with longer stance times on the prosthetic limb [7]. This modified motor pattern led to significant improvements both temporal and propulsive symmetry for a constant, self-selected walking speed [7]. While these results are promising, subjects in that study reported greater perceived difficulty with improved their gait symmetry. Individuals are less likely to maintain strategies that are more difficult, so this new walking pattern may be abandoned outside of the laboratory or clinic. Gait symmetry may be adversely affecting other important aspects of gait. Researchers have suggested that individuals with amputation prefer a longer duration on the intact limb (i.e. temporal asymmetry) because the intact limb has a greater ability to maintain balance [8]. Though, pain and discomfort in the residual limb may also be contributing factors. Understanding the effects of improved gait symmetry on balance control and associated effort in individuals with lower limb amputation may provide insight as to why symmetric gait is not generally preferred and identify potential adverse effects.

Frontal plane movement, particularly of one’s center-of-mass (COM) and center of pressure, have important roles in lateral balance during walking. Dynamic balance is defined here as the maintenance of the extrapolated center of mass (i.e. XCOM, a function of COM position and velocity) within the center of pressure of both limbs [9]. To reduce the likelihood of a loss of balance, Hof et al. proposed people use a simple control scheme, constant offset control, to maintain a safety margin (referred to as margin of stability) by consistently placing each foot a constant distance from the XCOM [10]. The most efficient method of preventing a loss of balance is a more lateral foot placement [11]. Indeed, in situations with larger expected perturbations, individuals typically increase their margin of stability by increasing stride width [12]. Similarly, individuals with lower limb amputation commonly exhibit larger stride width and margin of stability (particularly on the prosthesis side) compared to able-bodied people [13, 14], and it has been suggested that this behavior is attributable to the reduced stability of a prosthesis. However, wider-than-preferred stride widths have been associated with increased hip abductor activation [15] and metabolic cost [16]. Similarly, excessive lateral COM movement or trunk lean increases metabolic cost and perceived difficulty [17, 18], and lateral COM excursion is positively correlated with the metabolic cost of walking for individuals with TFA [19].

The objective of this study was to investigate the effects of increased stance time on the prosthesis, elicited by visual feedback, on the lateral balance and associated effort of individuals with TFA or KD walking with a powered knee prosthesis and no walking aid. We hypothesized that COM excursion would increase with longer stance time on the prosthesis, as slower cadence is typically associated with larger COM excursion [20]. We additionally hypothesized that the individuals would increase their stride width, at the cost of increased hip abductor activity, to preserve their margin of stability, in accordance with the constant offset control scheme and previous studies with able-bodied people and individuals with transtibial amputation [13]. While these populations are different from individuals with TFA and KD, we believe the concepts are applicable but potentially amplified in this population due to the loss of function from the knee joint. The additional demands for balance control in the frontal plane may contribute to the increased perceived difficulty with gait symmetry. Identification of these associated factors may inform interventions and/or development of assistive technology to reduce the effort associated with gait symmetry.

## Methods

### Participants

Our inclusion criteria were individuals with a unilateral TFA or KD who wore a prosthetic component daily, were able to walk independently without assistance (i.e. K-level 3 or 4), and were aged between 18 and 80. Our exclusion criteria were body weight greater than 165 kg (i.e. exceeding the limitation of Power Knee™ prosthesis) and any cognitive, vision, or cardiovascular complications that would affect the ability to complete our visual feedback protocol. We provided details of the six participants in Table 1; Subjects 1–5 are the same as those in [7]. All subjects reported that they wear their prescribed prosthesis daily for 12 or more hours, have had experience walking on a treadmill, and regularly participate in sports or activities besides walking. All subjects provided informed, written consent to participate in our protocol approved by The Institutional Review Board of the University of North Carolina at Chapel Hill, and we conducted our study in accordance with the relevant guidelines and regulations for human subjects.

**Table 1:** Participant Information. The prosthetic component weights listed correspond to the knee component only and do not include the weight of the socket or pylon. TFA corresponds to transfemoral amputation, and KD corresponds to knee disarticulation

Subject	1	2	3	4	5	6
Gender	Male	Male	Male	Male	Female	Male
Height	1.8 m	1.8 m	1.7 m	1.8 m	1.7 m	1.7 m
Body weight	69 kg	94 kg	61 kg	69 kg	49 kg	67 kg
Age	24 years	59 years	27 years	19 years	52 years	64 years
Time since amputation	7 years	47 years	4 years	19 years	27 years	17 years
Reason for amputation	Cancer	Cancer	Trauma	Congenital	Trauma	Trauma
Side of amputation	Right TFA	Left TFA	Right KD	Left TFA	Right TFA	Left TFA
Prescribed knee component	Genium (Ottobock, 1.7 kg)	Genium (Ottobock, 1.7 kg)	Plie 3 (Freedom Innovations, 1.2 kg)	Rheo Knee (Össur, 1.6 kg)	Power Knee (Össur, 3.2 kg)	C-Leg (Ottobock, 1.2 kg)
Prescribed ankle component	1C64 Triton Heavy Duty (Ottobock)	1E56 Axtion (Ottobock)	A low-profile high-performance foot	High-profile foot (Prosthetics Orthotics and Associates)	ProFlex (Össur)	ProFlex LP (Össur)
Prosthetic ankle component used with the powered knee component	ProFlex XC (Össur)	ProFlex XC (Össur)	A low-profile high-performance foot (due to long residual limb)	ProFlex XC (Össur)	ProFlex (Össur)	ProFlex XC (Össur)
Self-selected walking speed	0.8 m/s	0.8 m/s	0.7 m/s	0.7 m/s	0.5 m/s	0.9 m/s
Residual limb length	0.35 m	0.25 m	Long (KD)	0.39 m	Not available	0.39 m

**Table 2:** Temporal Measures

		None	L1	L2	L3	Percent Change	Feedback Main Effect		Effect Size
Stride time (seconds)	*prosthetic side*	1.33 (0.09)^bc^	1.31 (0.12)^c^	1.37 (0.12)^b^	1.43 (0.13)^a^	7%	***F*** **= 14.08**	***p*** **< 0.001**	**0.73**
	*intact side*	1.33 (0.09)^bc^	1.31 (0.12)^c^	1.37 (0.12)^b^	1.42 (0.13)^a^	7%	***F*** **= 13.77**	***p*** **< 0.001**	**0.73**
Stance time (seconds)	*prosthetic side*	0.83 (0.07)^b^	0.82 (0.08)^b^	0.85 (0.08)^b^	0.89 (0.09)^a^	7%	***F*** **= 13.60**	***p*** **< 0.001**	**0.73**
	*intact side*	0.94 (0.08)^b^	0.93 (0.09)^b^	0.96 (0.10)^ab^	0.98 (0.10)^a^	5%	***F*** **= 8.48**	***p*** **= 0.001**	**0.62**
Stance time asymmetry (%)		12.7 (6.3)^a^	12.9 (6.7)^a^	12.4 (7.8)^ab^	10.2 (7.3)^b^	−20%	***F*** **= 5.96**	***p*** **= 0.006**	**0.54**
Single support time/contralateral swing time (percent of stride)	*prosthetic side*	29.4 (2.8)^b^	29.2 (2.5)^b^	30.2 (2.5)^ab^	31.0 (2.5)^a^	5%	***F*** **= 7.32**	***p*** **= 0.003**	**0.59**
	*intact side*	37.8 (2.1)^a^	37.8 (2.5)^ab^	38.3 (2.9)^ab^	37.8 (2.7)^b^	0%	***F*** **= 2.60**	***p*** **= 0.085**	**0.35**
Double support time (percent of stride)	*prosthetic side trailing*	15.5 (1.3)^a^	15.6 (1.5)^a^	15.1 (1.4)^ab^	14.7 (1.1)^b^	−5%	***F*** **= 5.64**	***p*** **= 0.008**	**0.53**
	*intact side trailing*	17.3 (2.6)	17.4 (2.7)	16.5 (3.0)	16.5 (2.6)	−5%	***F*** = 2.10	*p* = 0.141	

The first four columns summarize the group mean and standard deviation of the testing conditions. The fifth column is the percent change between the first and last condition, i.e. None and L3. The sixth column is the one-way ANOVA results for the main effect of feedback, with bold font denoting statistical significance *p* < 0.1. Post-hoc multiple comparisons results are denoted by superscript lettering, with conditions not sharing the same lettering being statistically different. The last column is the effect size (partial eta squared) of the visual feedback for statistically significant changes

### Experimental setup

All subjects visited the lab three times for 1) fitting and training with the powered prosthesis and walking sessions with their 2) prescribed prosthesis and 3) powered prosthesis, and only the data collected in the last visit (powered prosthesis testing) was used for this study. Thus, subjects (except for Subject 5) only walked with the powered prosthesis for a total of two sessions. All subjects performed the walking session (with and without visual feedback, as described in the experimental design section) with their prescribed device first to ensure familiarity with the visual feedback paradigm.

A certified prosthetist, who completed training specific for the alignment and fitting of the Power Knee™, aligned and tuned the Power Knee™ prosthesis for Subjects 1–4 and 6 prior to testing. Subject 5 uses the same device daily, so we maintained her alignment and settings as set by her certified prosthetist.

During testing, all subjects walked at their self-selected speed on a treadmill with the commercially-available Power Knee™ prosthesis (Össur, Reykjavík, Iceland). The handrails remained on the treadmill at all times for subjects’ safety, but we encouraged subjects not to rely on them during testing. In order to investigate lateral balance control, it was crucial that subjects did not walk with assistance (e.g. touching treadmill handrails) during the strides selected for analysis.

### Visual feedback

We created a real-time visual feedback display in MATLAB (The MathWorks, Inc., Natick, Massachusetts, USA) using Vicon DataStream SDK (VICON, Oxford, UK), as described in [7]. We displayed the visual feedback at eye level on a 0.6-m computer monitor located 1 m in front of the treadmill. Our custom MATLAB script 1) extracted the vertical ground reaction force from the side of the treadmill corresponding to the subject’s amputated limb, 2) calculated stance time based on the length of time a vertical force greater than 10% of the subject’s body weight was detected (a high threshold value was used in case of sensor drifting or noise), 3) averaged the stance time of the subjects’ previous five strides to smooth the signal, 4) displayed this averaged stance time value as a large, blue dot in the horizontal center of the computer screen, and 5) updated this value after every toe-off event of the corresponding limb. The blue dot moved vertically upward along the y-axis as subjects increased stance time of the corresponding limb. A horizontal, grey line across the entire screen represented the subject’s target. It remained in the center of the screen for all conditions to prevent subject bias from confounding our results. The y-axis of the display remained 0.2 s above and below the target line to maintain subjects’ perceived accuracy. We selected the parameters of the display through iterations of pilot testing based on their simplicity and usability across multiple people.

### Experimental design

To find each subject’s self-selected speed at the beginning of each testing session, we increased/decreased the speed three times by 1 m/s increments until the subject stated that the speed was too fast/slow. We recorded and then averaged the six speeds that were too fast/slow [21]. We provided time for subjects to practice using the visual feedback, but we kept this time minimal to prevent fatigue in our testing trials. Likely because we used a simple metric and display, all subjects learned how to modulate the blue dot/stance time very quickly (i.e. within 30 s).

During testing, all subjects completed twelve 1.5-min walking trials. We administered 2 minutes of rest between trials and longer periods of rest if the subject or researchers detected any fatigue. We randomized the four conditions (i.e. no feedback, visual feedback target Level 1, Level 2, and Level 3) within three testing periods to prevent training or fatigue from confounding our results. The first trial was always a no-feedback trial (i.e. baseline) in order to determine the subsequent visual feedback target levels:

- Level 1 (L1): preferred stance time of his/her amputated limb from the baseline trial
- Level 2 (L2): mid-point between Level 1 and Level 3
- Level 3 (L3): preferred stance time of his/her intact limb from the baseline trial

The visual feedback level targets remained constant throughout each trial and testing day. Real-time feedback was only provided for the amputated limb.

### Measurements

We recorded the ground reaction forces of each limb from a dual-belt treadmill (1000 Hz, Bertec Corp., Columbus, OH, USA), full-body motion using a 12-camera motion capture system and 39 retroreflective markers (100 Hz, VICON, Oxford, UK), and *gluteus medius* activity of each limb using active surface electromyography (EMG) sensors (Sensor SX230, Amplifier K800, Biometrics Ltd., Ladysmith, VA, USA). The markers on the prosthesis-side were placed on the estimated axes of rotation [22]. We prepared the skin and electrodes using isopropyl alcohol. We placed the EMG sensors in accordance with the SENIAM standards, and we placed the ground electrode on a bony landmark near the wrist. We verified the location of the sensors and signal quality by visually examining the EMG signal as subjects placed their hands on a table in front of them and abducted each leg individually. Though it was not part of our main objective, we also recorded subjects’ verbal feedback regarding their experience with the powered prosthetic knee component.

### Data processing

We selected nine consecutive strides from each trial, as it was the maximum number of consecutive strides in which subjects did not touch handrails or scuff the treadmill belts. Subject 5 touched the handrails more frequently, and we were only able to extract nine, clean strides for (and subsequently only included) ten of the twelve total trials. We low-pass filtered (Butterworth, 4th order, 7.5 Hz) the ground reaction force and motion data. We baseline-corrected the ground reaction force data and used a 20 N threshold to identify gait events. We manually calculated all temporal parameters using these gait events. We used Visual 3D software (C-Motion, Inc., Germantown, MD, USA) to calculate COM position and velocity after manually adjusting for the inertial properties of the prosthesis segments. For the EMG signals, we high-pass filtered (Butterworth, 20 Hz, 4th order), full-wave rectified, and low-pass filtered (Butterworth, 10 Hz, 4th order) them to create a linear envelope. We then normalized the envelope to the peak amplitude across all strides [23] and calculated the integral as a proxy for muscle effort.

We defined stride width to be the medial-lateral distance between the subject’s heels at ipsilateral heel strike and the subsequent contralateral heel strike. Medial-lateral COM excursion is defined as the maximal displacement of the COM in the frontal plane during a stride, where maximal lateral positioning occurred during single-support phase of each limb. As a measure of the quality of balance, we calculated the minimum distance between subjects’ XCOM and COP, referred to as the margin of stability [9]. We used the equation for XCOM that Hof et al. developed and applied with individuals with TFA [14]:

$$ XCOM(t)=z(t)+\frac{1}{\sqrt{g/h}}\cdotp \frac{dz}{dt} $$

where *z* is the lateral position of the COM (m) (positive to the right), *g* is gravitational acceleration, and *h* is 1.34 times leg length (m), which was calculated as the average of the two trochanters. The minimum margin between the XCOM and COP occurred near heel strike of each limb.

### Statistical tests

Using the average of the three replicate trials for each testing condition, we used repeated-measures ANOVA to test for significant effects of the visual feedback. We included testing period as a fixed blocking factor and subject and its interaction with visual feedback as random blocking factors. We set alpha at 0.01 and reported all F- and *p*-values for reference. We used the Shapiro-Wilk normality test (*p* < 0.01) to detect outlier trials [24]. In total, we identified five outlier trials, but we excluded only the two trials that were outliers across multiple responses. The inclusion/exclusion of any of these trials did not affect the significance of our results (Additional file 1: Table S1). The removal of more trials may have removed valuable information from the study. When we found a significant main effect, we used Tukey’s honestly significant difference test (alpha = 0.1) to test for statistical difference between conditions. We reported effect sizes as partial eta-squared (${\eta}_p^2$):

$$ {\eta}_p^2=\frac{SS_{effect}}{SS_{effect}+{SS}_{effect\ast subject}} $$

where SS is the sum of squares, and effect is the effect of visual feedback. To further investigate the linear relationship between stance time and margin of stability on the prosthetic limb, we calculated the Pearson’s correlation coefficient for each subject.

**Table 3:** Lateral Balance Measures

		None	L1	L2	L3	Percent Change	Feedback Main Effect		Effect Size
Lateral margin of stability (cm)	*prosthetic side*	5.71 (1.18)^ab^	5.91 (1.02)^a^	5.61 (1.31)^bc^	5.30 (1.15)^c^	−7%	***F*** **= 12.31**	***p*** **< 0.001**	**0.71**
	*intact side*	4.92 (1.18)	5.02 (1.26)	4.79 (1.29)	4.65 (1.17)	−5%	*F* = 2.07	*p* = 0.145	
Stride width (cm)		19.4 (2.6)	19.7 (2.3)	19.9 (2.9)	20.5 (3.2)	6%	*F* = 0.59	*p* = 0.634	
M-L COM excursion (cm)		5.96 (0.83)^b^	5.97 (0.89)^b^	6.58 (0.97)^ab^	7.05 (1.22)^a^	18%	***F*** **= 4.22**	***p*** **= 0.024**	**0.45**
Hip abductor activity (normalized, integrated EMG)	*prosthetic side*	0.17 (0.07)^b^	0.17 (0.06)^b^	0.19 (0.07)^ab^	0.21 (0.06)^a^	25%	***F*** **= 6.69**	***p*** **= 0.004**	**0.57**
	*intact side*	0.17 (0.11)^ab^	0.16 (0.10)^b^	0.19 (0.12)^ab^	0.20 (0.13)^a^	18%	***F*** **= 2.83**	***p*** **= 0.071**	**0.36**

The first four columns summarize the group mean and standard deviation of the testing conditions. The fifth column is the percent change between the first and last condition, i.e. None and L3. The sixth column is the one-way ANOVA results for the main effect of feedback, with bold font denoting statistical significance *p* < 0.1. Post-hoc multiple comparisons results are denoted by superscript lettering, with conditions not sharing the same lettering being statistically different. The last column is the effect size (partial eta squared) of the visual feedback for statistically significant changes

## Results

Subjects walked at their self-selected speeds during testing (Table 1). With visual feedback, subjects increased their stride time (*p* < 0.001, ${\eta}_p^2$ =0.73 both limbs) (Table 2). Compared to the no-feedback condition, the highest feedback level (L3) elicited an increase in stance time of 7% on the prosthesis side and 5% on the intact side. Thus, stance time symmetry significantly improved by 20% (*p* = 0.006, ${\eta}_p^2$ =0.54) (Table 2). On the prosthesis side, single support time as a percentage of stride time significantly increased (*p* = 0.003, ${\eta}_p^2$ =0.59), and terminal double support time significantly decreased (*p* = 0.008, ${\eta}_p^2$ =0.53) (Table 2).

**Fig. 1:**
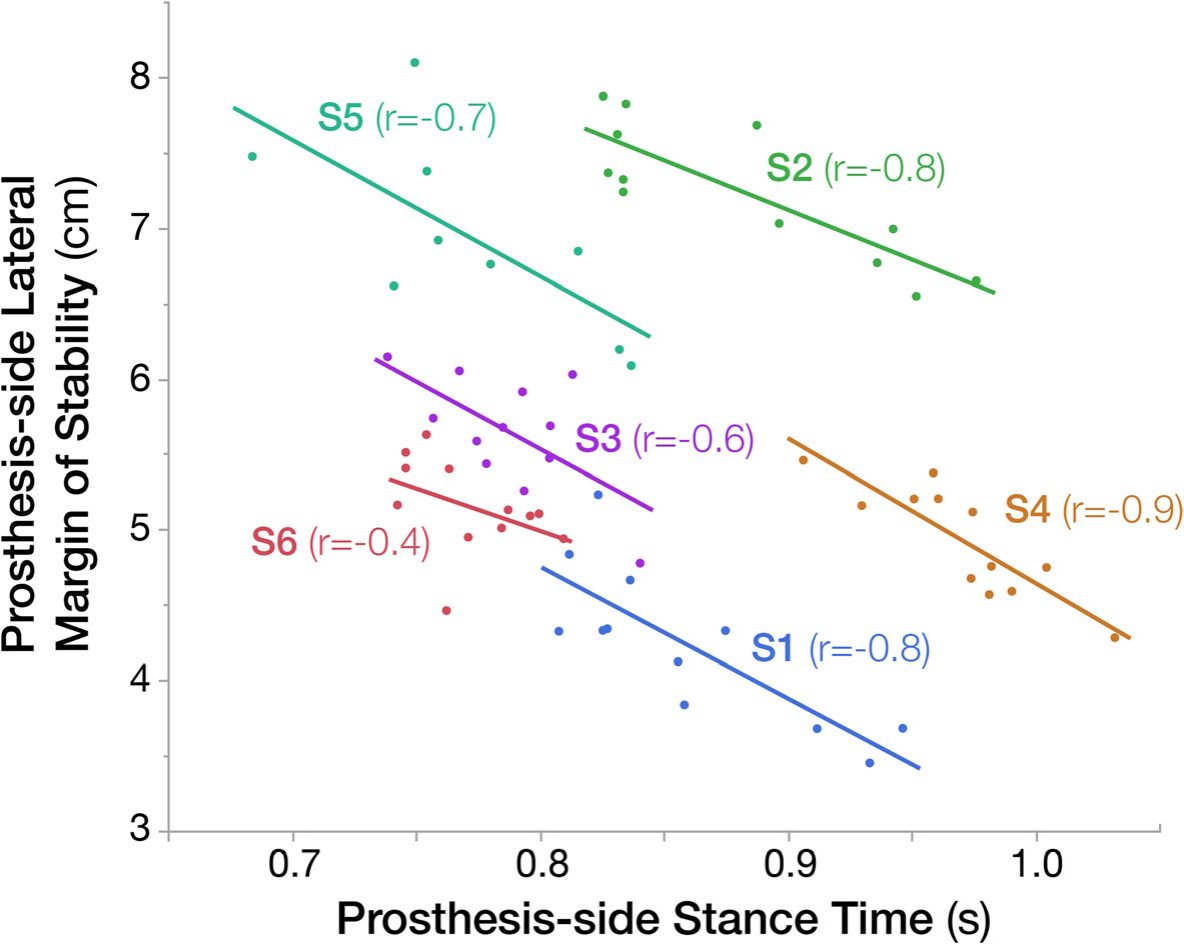
Prosthesis-side lateral margin of stability was negatively correlated with prosthesis-side stance time across subjects. Point characters indicate the average of 9 strides from each trial, with a linear regression line to demonstrate the inverse relationship. Correlation coefficients between prosthesis-side stance time and prosthesis-side lateral margin of stability shown for each subject (S1-S5). Color of point characters, lines, and correlation coefficients correspond to each subject

Medial-lateral COM excursion significantly increased with feedback (*p* = 0.024, ${\eta}_p^2$ =0.45), and the lateral margin of stability significantly decreased on the prosthesis side only (*p* < 0.001, ${\eta}_p^2$ =0.71) (Table 3). Stride width did not significantly differ between conditions (*p* = 0.634) (Table 3). Prosthesis-side lateral margin of stability negatively correlated with prosthesis-side stance time, with correlation coefficients ranging − 0.4 to − 0.9 for each subject (Fig. 1).

**Fig. 2:**
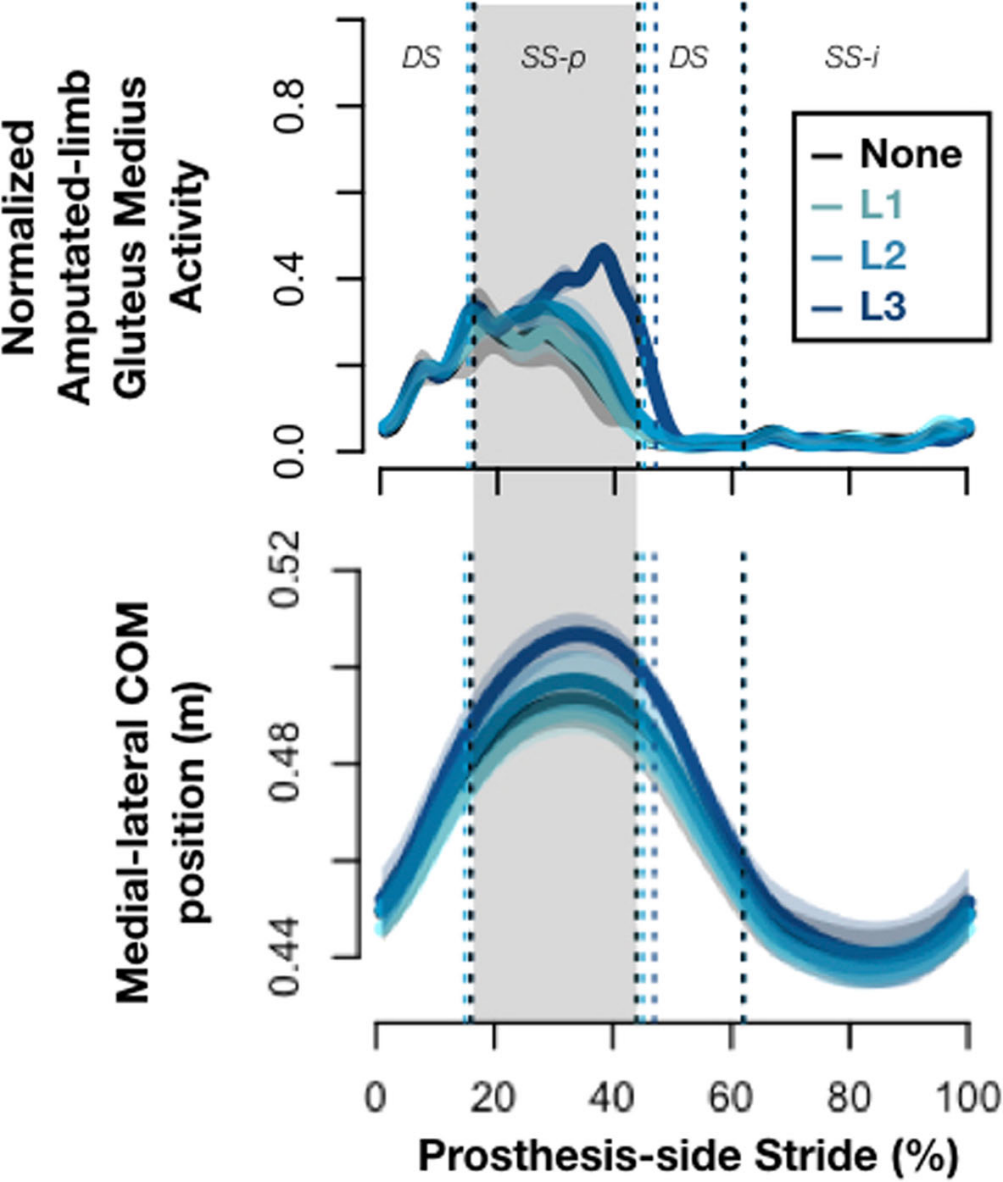
A more lateral COM position was associated with increased hip abductor activation. Normalized amputated-limb *gluteus medius* activity and medial-lateral COM position shown for one representative subject (S1), normalized to stride time. Mean and one standard deviation are represented by the solid line and lighter shaded area around the solid line, respectively. Colors represent each testing condition: no feedback (None) and levels 1–3 of the visual feedback (L1–3). Level 3 corresponds to the longest prosthesis-side stance time. Vertical dotted lines indicate gait events: (from left to right) contralateral toe off, contralateral heel stride, and ipsilateral toe off. Each gait phase is labeled: double-support (DS), single-support prosthesis side (SS-p), and single-support intact side (SS-i). Single-support phase is shaded grey to highlight the gait phase in which the greatest changes occurred with visual feedback

**Fig. 3:**
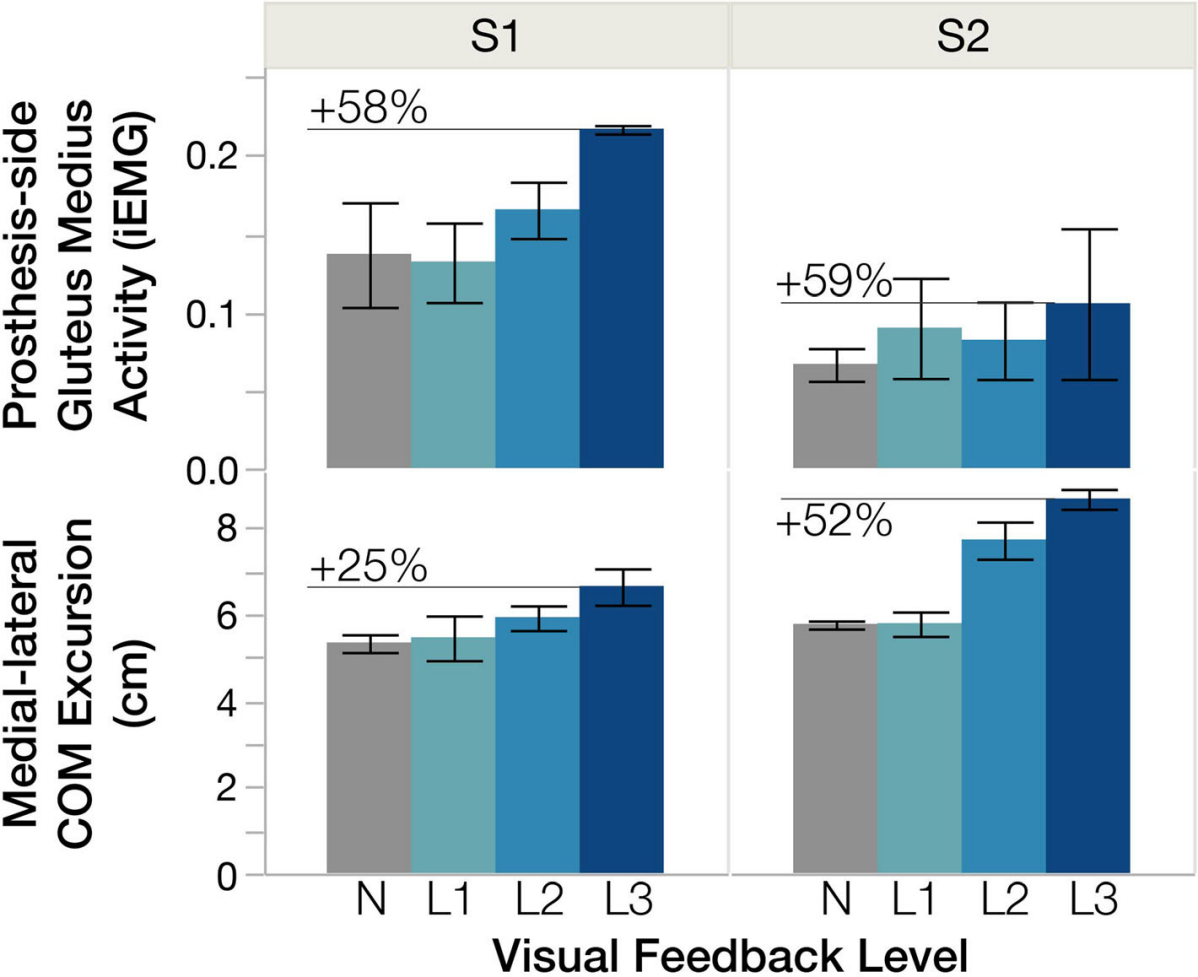
A similar increase in amputated-limb hip abductor activity did not correspond to a similar change in medial-lateral COM excursion. Integrated amputated-limb *gluteus medius* activity and medial-lateral COM excursion shown for two representative subjects (S1, S2). Bars indicate the mean and error bars indicate one standard deviation from the mean. Colors represent each testing condition: no feedback (N) and levels 1–3 of the visual feedback (L1–3). Level 3 corresponds to the longest prosthesis-side stance time. Percent change between the no-feedback and visual feedback level 3 are labeled

*Gluteus medius* activity increased during stance phase, particularly during single-support phase in which the COM moved more laterally, with a greater change occurring on the prosthesis side (e.g. Figure 2). Integrated EMG of the *gluteus medius* significantly increased with visual feedback, with a larger percent change on the prosthesis side (Table 3) (*p* = 0.004, ${\eta}_p^2$ =0.57 prosthetic side, *p* = 0.071, ${\eta}_p^2$ =0.36 intact side). Increasing prosthesis-side *gluteus medius* activity had a variable relationship with subjects’ COM excursion, as demonstrated in Fig. 3 with two representative subjects.

During testing, Subject 1 described the powered knee as “more natural”, “more like a part of me”, and that it “acts more like a muscle” compared with his prescribed device. In contrast, Subject 2 did not enjoy walking with the powered prosthesis. Subject 5 spoke highly of the functionality of the device, as it is her daily and preferred device. The remaining subjects did not seem to have strong opinions but noted the weight of the device.

## Discussion

To our knowledge, this is the first study to investigate the effects of increased stance time (and improved stance time symmetry) on lateral COM movement and balance in individuals with TFA or KD in use of a powered prosthetic knee component. In partial support of our hypotheses, increased stance time on the prosthesis via visual feedback yielded increased medial-lateral COM excursion and hip abductor activity. Unexpectedly, stride width did not significantly increase with visual feedback, and prosthesis-side lateral margin of stability significantly decreased. Unfortunately, the efforts related to maintaining lateral balance in individuals with TFA or KD may be limiting them from reaching a more symmetric gait pattern on their own. Based on these results, we strongly believe sagittal-plane and temporal measures are not sufficient evaluation metrics when considering the usability and translation of advanced prostheses from the laboratory to daily use.

The margin of stability is directly related to the minimum amount of impulse one can handle before losing balance [9], so walking with a smaller margin is interpreted as less stable. Increased COM excursion likely contributed to the reduction in stability on the prosthetic side. Though we expected the individuals with TFA or KD to increase their stride width sufficiently to prevent this decline in stability, there may be many possible explanations for these results. First, maintaining a more stable posture may have conflicted with other objectives of walking, such as energy cost. Second, residual muscle impairments and reduced sensory feedback on the prosthesis side may have limited their ability to walk with a more stable pattern. The hip abductor muscles, *glutei medii* in particular, are used to stabilize the pelvis during stance phase, but they often lose volume and atrophy post amputation, depending on the type of surgery [25]. It may not have been feasible for subjects to further increase activation of their *gluteus medius* on the amputated side, in order to increase stride width and/or stabilize the COM. As an example, Subjects 1 and 2 increased their prosthesis-side *gluteus medius* activity by a similar amount with increased prosthesis-side stance time, but Subject 2 exhibited considerably larger COM excursions compared to Subject 1 (Fig. 3). Many factors contribute to inter-subject differences, including residual limb length, surgery type, and muscle atrophy post amputation. During testing, Subject 1 spoke favorably of the powered prosthesis, while Subject 2 did not. These subjective differences may be linked to the difference in COM excursion or effort required to maintain balance during testing.

Moreover, a lateral COM shift or trunk lean toward the prosthesis-side is a common compensatory movement for individuals with TFA [26], and may have contributed to larger changes in stability on the prosthesis side. This compensatory movement is thought to reduce pressure on the medial-lateral aspects of the residual limb from the socket and reduce hip abductor demands, as abnormal loading on the residual-limb soft tissues has been suggested as a factor contributing to their perception of discomfort [27]. However, a more active trunk movement strategy, common among individuals with TFA, has been shown to increase frontal-plane joint powers in the low back, potentially contributing to low back pain that is prevalent in individuals with lower limb amputation [28].

Lastly, the decrease in the subjects’ margin of stability may be associated with their decrease in stride frequency (evident by increased stride time at a constant treadmill speed). Researchers have demonstrated that the lateral margin of stability is positively correlated with stride frequency in able-bodied people [29], but this relationship alone does not explain the larger changes in stability on the subjects’ prosthesis side compared with their intact side. Instead, a combination of the explanations above (e.g. muscle atrophy combined with lateral trunk lean) likely contributed to the results in this study.

### Potential solutions

Interventions aimed at improving the lateral balance of individuals with TFA or KD may decrease the difficulty of walking more symmetrically with a powered prosthesis. Physical therapists often recommend hip abduction exercises during the rehabilitation process to improve the balance of individuals with lower limb amputation. However, exercises alone may not be sufficient, depending on the individual’s muscular changes post amputation. We provided additional considerations below for improved prosthesis control and/or assistive devices.

First, evidence suggests a relationship between propulsion and stability. One study demonstrated that able-bodied people prefer a propulsive force that maximizes dynamic stability [30]. Another group improved the step width variability and metabolic rate of individuals using a powered ankle prosthesis by modulating ankle push-off in response to COM lateral velocity [31]. “Push-off strategy” is even considered a strategy employed to control dynamic balance [10], similar to weight transfer during standing. Modulating prosthesis push-off control for individuals with TFA or KD may also be a feasible solution. For example, using the control in [31] in a powered ankle coupled with a powered knee may be one method. But, modulating ankle push-off in coordination with control of the knee will be an important challenge to overcome, as the knee joint plays a role in both propulsion and balance as well [32]. Real-time, automatic adjustments to powered knee parameters in response to the user and prosthesis states has been demonstrated [33, 34], and may be required to maintain proper knee dynamics (e.g. extension torque during propulsion) with changing ankle dynamics.

Second, ankle strategy (i.e. inversion/eversion) is another balance strategy used to adjust the base of support or center of pressure [14]. Recent advances in prosthesis control have demonstrated inversion/eversion is now a possibility for powered prosthetic ankles, and may have implications for lateral balance [35]. However, previous research demonstrates that additional mass to the lower limb can negatively affect individuals’ lateral stability [36], with distal weight more destabilizing than proximal weight. This relationship should be an important consideration in the development of powered knee-ankle prostheses that have a second, distally-located motor.

Last, external stabilization may also reduce the challenges of walking with improved propulsive symmetry. External stabilization via cables elicited a decrease in stride width and metabolic cost in able-bodied people [37]. Additionally, a hip-stabilizing brace enhanced the dynamic stability of individuals with weak hip abductor muscles [38].

Visual feedback can be used as a training tool in rehabilitation to encourage and reinforce more efficient motor patterns that are maintained after the visual feedback is removed. For instance, in a 3-week long case study, one individual with TFA maintained improved hip and trunk kinematics after visual feedback (and therapist feedback) were removed [39]. Though we did not study training here, we would not expect individuals with amputation to maintain temporal symmetry if we removed our visual feedback, due to the added cost of maintaining balance. Perhaps training with a combination of tools (e.g. improved prosthesis control/stabilizing brace in combination with visual feedback) will yield more favorable results after feedback is removed. We have yet to understand the long-term effects of these approaches and the interaction between them. Furthermore, it has been demonstrated that voluntary correction of gait patterns is a separate mechanism from learning, and the intersection of these two mechanisms remains an important area of gait rehabilitation research [40].

### Limitations

The small sample size of this study limits the generalizability of our results to the entire population of individuals with lower limb amputation. However, due to the small sample size and exploratory nature of this analysis, we chose an alpha level of 0.1. We believe a Type II error has an equal if not a more severe consequence in this study, and a less conservative alpha level compared to 0.05 allows us to reduce the probability of Type II error to more evenly balance the probability of incurring Type I and Type II errors [41]. Moreover, we hope that researchers will continue to supplement their data with individual subject results and interpretations, in order to aid clinicians and engineers in personalizing treatments/solutions for individual patients. Inter-subject differences in this study may be attributed to many factors, including self-selected speed, surgical methods, residual-limb length and musculature, and overall walking function.

Another limitation of this study is that we did not use the same prosthetic ankle component across all subjects; the ProFlex XC ankle component is recommended for use with the Power Knee, but Subject 3 required a low-profile foot due to his long residual limb, and Subject 5 maintained her daily components and alignment to preserve natural behavior. Using the same powered prosthetic knee component across individuals allowed us to reduce confounding effects associated with prosthetic knee joint mechanics, but future studies comparing individuals’ balance between powered and passive (e.g., mechanical, microprocessor) knee prostheses may provide additional insight for the advancement of prosthetic knees.

Additionally, studies have demonstrated that prosthesis control parameters can influence gait symmetry [42]. While we held control parameters constant to reduce confounding effects in this study, alternate parameters may be more appropriate once the user adopts a more symmetric gait strategy, warranting further investigation. Subjects’ limited training time with the powered device (e.g. 2 days) may not be sufficient to generate optimal behavior.

## Conclusion

Establishing gait symmetry is often a rehabilitation goal in order to preserve the intact limb. However, with longer stance time on a powered knee prosthesis and an improvement in stance time symmetry, we observed a decrease in the prosthesis-side lateral margin of stability. Our results suggest subjects’ preference in walking with less stance time on the prosthesis is associated with reduced effort to maintain lateral balance. With improved control of lateral balance (i.e. COM dynamics, foot placement, or a combination of both), individuals with TFA or KD may more easily maintain gait symmetry in the long term. Balance in particular is of high importance for individuals when using their prosthesis [43], and should be an important consideration in prosthesis control and training.

## Supplementary information

**Additional file 1: Table S1.** ANOVA results with the inclusion/exclusion of each identified outlier trial. In total, we identified five outlier trials in five outcome measures (listed below). However, the inclusion/exclusion of these identified outlier trials did not affect the significance of our results (below, significance level 0.01). Therefore, we only excluded two outlier trials that were outliers in multiple responses (i.e. one outlier trial in stride time and stance time, one outlier trial in swing time and double support time).

## Data Availability

The data from this study is available from the corresponding author upon reasonable request.

## Abbreviations

L1–3: Level 1–3 of the visual feedback
None: No feedback condition
