# Supplementary material for: Effects of extended stance time on a powered knee prosthesis and gait symmetry on the lateral control of balance during walking in individuals with unilateral amputation
Source: J Neuroeng Rehabil. 2019 Nov 29;16:151. doi: 10.1186/s12984-019-0625-6 (PMC6883569; doi:10.1186/s12984-019-0625-6)
Supplement: Supplementary file 1 — Additional file 1: Table S1. ANOVA results with the inclusion/exclusion of each identified outlier trial. In total, we identified five outlier trials in five outcome measures (listed below). However, the inclusion/exclusion of these identified outlier trials did not affect the significance of our results (below, significance level 0.01). Therefore, we only excluded two outlier trials that were outliers in multiple responses (i.e. one outlier trial in stride time and stance time, one outlier trial in swing time and double support time). [file 12984_2019_625_MOESM1_ESM.docx]

| **Outcome measure** | **Included/excluded outlier trial(s)** | **Feedback**  **main effect** |
| --- | --- | --- |
| **Stride time - intact side** (2 trials) | *Included* | 0.001 |
|  | *Excluded* | <0.001 |
| **Stance time - intact side** (same as one trial from stride time) | *Included* | 0.007 |
|  | *Excluded* | 0.002 |
| **Swing time - prosthesis side** (1 trial) | *Included* | 0.030 |
|  | *Excluded* | 0.014 |
| **Double support time - intact side leading** (same trial as swing time) | *Included* | 0.040 |
|  | *Excluded* | 0.015 |
| **Gluteus medius activity - intact side** (2 independent trials) | *Included* | 0.056 |
|  | *Excluded* | 0.036 |
